# ﻿*Ligulariamonocephala* (Asteraceae, Senecioneae), a remarkable new species from Hubei, China

**DOI:** 10.3897/phytokeys.189.80016

**Published:** 2022-01-28

**Authors:** Wen-Qun Fei, Tao Deng, Long Wang

**Affiliations:** 1 Key Laboratory of Plant Resources Conservation and Sustainable Utilization, South China Botanical Garden, Chinese Academy of Sciences, Guangzhou 510650, Guangdong, China South China Botanical Garden, Chinese Academy of Sciences Guangzhou China; 2 University of Chinese Academy of Sciences, Beijing 100049, China University of Chinese Academy of Sciences Beijing China; 3 Key Laboratory for Plant Diversity and Biogeography of East Asia, Kunming Institute of Botany, Chinese Academy of Sciences, Kunming 650201, Yunnan, China Kunming Institute of Botany, Chinese Academy of Sciences Kunming China

**Keywords:** Compositae, Hubei, Shennongjia, taxonomy

## Abstract

*Ligulariamonocephala*, a remarkable new species from Hubei, China, is described and illustrated. It is readily distinguishable in the whole genus by character combination of the reniform to cordate-reniform leaf blades which are palmately-pinnately veined and abaxially purplish red, the solitary and erect capitula, and the pappus which are as long as or slightly longer than tube of the tubular corolla. A detailed description and distribution map of the species are also presented herein.

## ﻿Introduction

*Ligularia* Cass., as the largest genus of tribe Senecioneae in Asteraceae in China, consists of approximately 130 species distributed mainly in eastern Asia (Liu 1989; Liu et al. 1994; Nordenstam 2007; Nordenstam et al. 2009; Liu and Illarionova 2011; Guo and Wang 2021). Since the publication of *Flora of China* (Liu and Illarionova 2011), the updated English version of *Florae Reipublicae Popularis Sinicae* (Liu 1989), extensive works have been done to the taxonomy of this genus at specific level (Fei et al. 2019; Lazkov and Sennikov 2019; Guo and Wang 2021, and references therein).

During a field expedition in August 2016 to Shennongjia, Hubei, China, the second author was able to discover a unique *Ligularia* population in an alpine region of this area. The plant, at first sight, shows an affinity with *Ligulariahookeri* (C.B. Clarke) Hand.-Mazz. in habit, however, the leaf color and the capitula orientation can easily set them apart. An in-depth survey of herbarium specimens was conducted, resulting in the finding of four gatherings (*Anonymous 662*, *D.G. Zhang 080826018*, *Z.E. Zhao 1609* and *X.L. Yu et al. 080078*) all made from Shennongjia, Hubei, China, that are morphologically in conformity with this plant. To precisely decide the identity of these gatherings, we conducted another two field investigations to Shennongjia in September, 2020 and August, 2021, respectively, leading to a better understanding of the variation range of several main morphological characters of this plant. Upon careful observations and comparisons, it was found to be quite different from any other species in the genus in an array of morphological characters. We therefore concluded that this plant represents a hitherto undescribed species, which we describe below.

## ﻿Materials and methods

For morphological comparisons, we critically examined physical or digitalized herbarium specimens with high-resolution of the genus *Ligularia* at A, BM, CDBI, CSFI, E, GH, HIB, HITBC, HNWP, IBSC, JIU, K, KATH, KUN, LE, NAS, NY, P, PE, S, SZ, W, WU, and WUK (acronyms follow Thiers (2021)). Specimens were collected from Hubei (first in September 2020, second in August 2021) during our several field expeditions. The morphological measurements in the description are based on the *in-situ* observations and dried specimens deposited at IBSC and KUN. Its records of distribution, habitat and phenology are based on both field investigations and specimen records.

## ﻿Taxonomy

### ﻿ Ligularia monocephala

Taxon classificationPlantaeAsteralesAsteraceae

Long Wang sp. nov.

F232615C-22B6-5B7E-B330-C82796BC45B6

urn:lsid:ipni.org:names:77254167-1

Figures 1 , 2

#### Diagnosis.

Readily distinguishable in the whole genus by the character combination of the reniform to cordate-reniform leaf blades which are purplish red abaxially and palmately-pinnately veined, the solitary and erect capitula, and the pappus which are as long as, or slightly longer, than tube of the tubular corolla.

**Figure 1.**
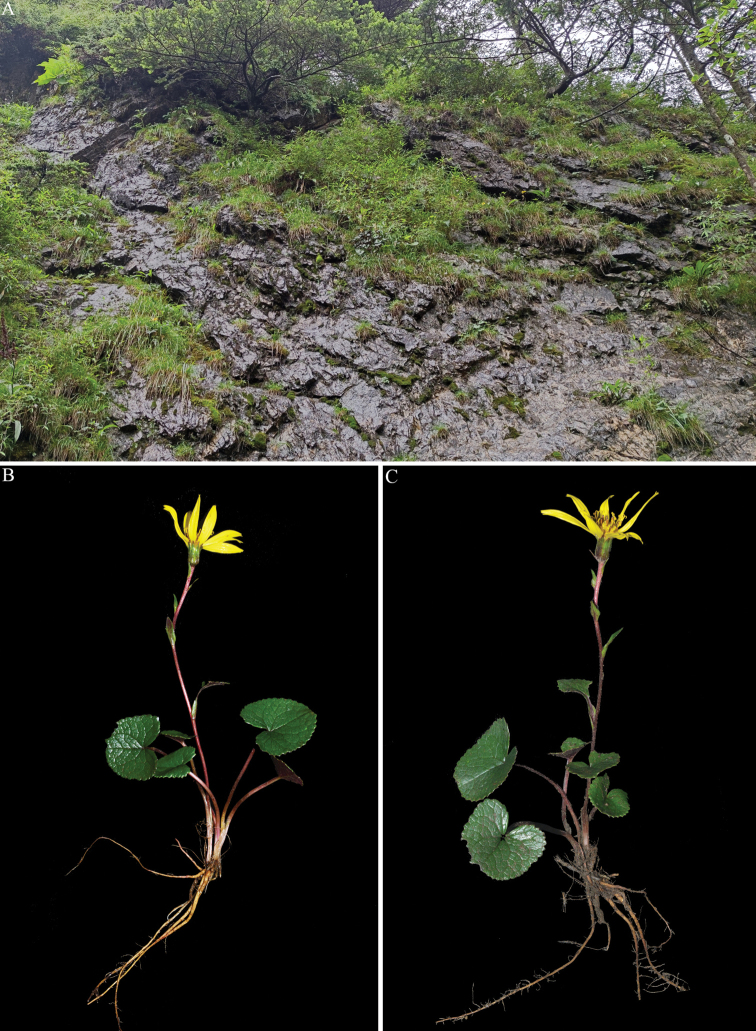
Photographs of *Ligulariamonocephala* sp. nov. **A** habitat **B, C** habit. All photographs by Wen-Qun Fei.

#### Type.

China. Hubei Province, Shennongjia Forest Department, Shennongding Nature Reserve, Shennonggu valley, 31°26'19.36"N, 110°16'26.46"E, 2681 m a.s.l., on cliffs, 12 July 2021, *W.Q. Fei & H.S. Wu 324* (holotype: IBSC; isotypes: IBSC, KUN).

**Figure 2.**
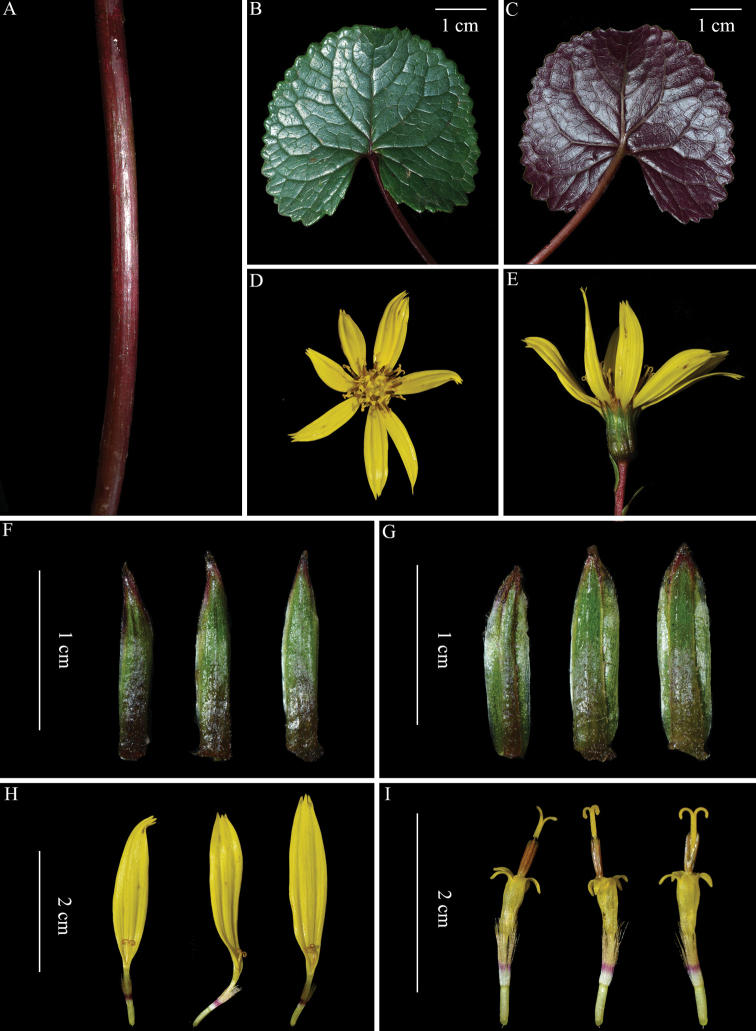
Photographs of *Ligulariamonocephala* sp. nov. **A** portion of stem **B** leaf blade (adaxial surface) **C** leaf blade (abaxial surface) **D** capitulum (top view) **E** capitulum (side view) **F** outer phyllaries (abaxial surface) **G** inner phyllaries (abaxial surface) **H** ray florets **I** tubular florets. All photographs by Wen-Qun Fei.

#### Description.

Perennial herb. Stems solitary, erect, 15–20 cm tall, ca. 3 mm in diam. at base, proximal to median part slightly brown puberulent and white arachnoid to glabrescent, distal part densely brownish pilose. Basal leaves ascending, long petiolate; petiole 5–10 cm long, not winged throughout; leaf blade reniform to cordate-reniform, 3.5–4.5(–6) cm long, 3.5–5(–7) cm wide, herbaceous, adaxially dark green, slightly shortly puberulent to glabrescent, abaxially purplish red, brownish puberulent at first, especially along veins, then becoming glabrescent, base cordate, margin regularly dentate, apex rounded or slightly obtuse; sinus narrow, basal lobes nearly rounded, slightly divergent; vein palmate-pinnate; primary veins 3–5. Stem leaves 4–6(–7). Proximal stem leaves usually 1, petiolate; petioles ca. 3 cm long, basally sheathed; sheath usually more or less broadened; leaf blade reniform to cordate-reniform, slightly smaller than basal leaves. Median stem leaves usually 1, nearly sessile; leaf blade usually less than 3 cm long and 3 cm wide; base slightly or enlarged sheathed. Distal stem leaves usually 2–5, much smaller and reduced, bract-like, lanceolate, 6–10 mm long, 1.5–3 mm wide; margin slightly ciliate or entire. Capitula solitary, erect, 5 cm in diam. including ray florets; bracts 1 or 2, lanceolate to subulate, 3–4 mm long, ca. 1 mm wide. Involucres cylindrical, 11–14 mm high, 9–14 mm in diam., outside shortly brownish puberulent; receptacle densely shortly puberulent outside; phyllaries 9–10, spreading, in 2 rows; outer phyllaries narrowly oblong, 2–2.5 mm wide, apex acute; inner phyllaries oblong, ca. 3 mm wide, margin membranous, apex acute to obtuse. Ray florets 6–9, yellow; lamina oblong to elliptic, 3.0–3.5 cm long, 6–7 mm wide, apex acute, 3-denticulate; tube 5 mm long. Tubular florets numerous, yellow, ca. 2 cm long; tube 5 mm long; limb campanulate, 5–7 mm long; style 1.2 cm long. Achenes (immature) oblong, cylindrical, pale yellow, 3–4 mm long, glabrous. Pappus brown in the upper two-thirds, white (distal part) and purplish red (proximal part) in the lower one-third, 5–7 mm long, as long as, or slightly longer, than tube of tubular corolla.

#### Etymology.

The specific epithet ‘*monocephala*’ alludes to occurrence of solitary capitulum per stem.

#### Phenology.

Flowering from July to August; fruiting in September.

#### Distribution and habitat.

**Figure 3.**
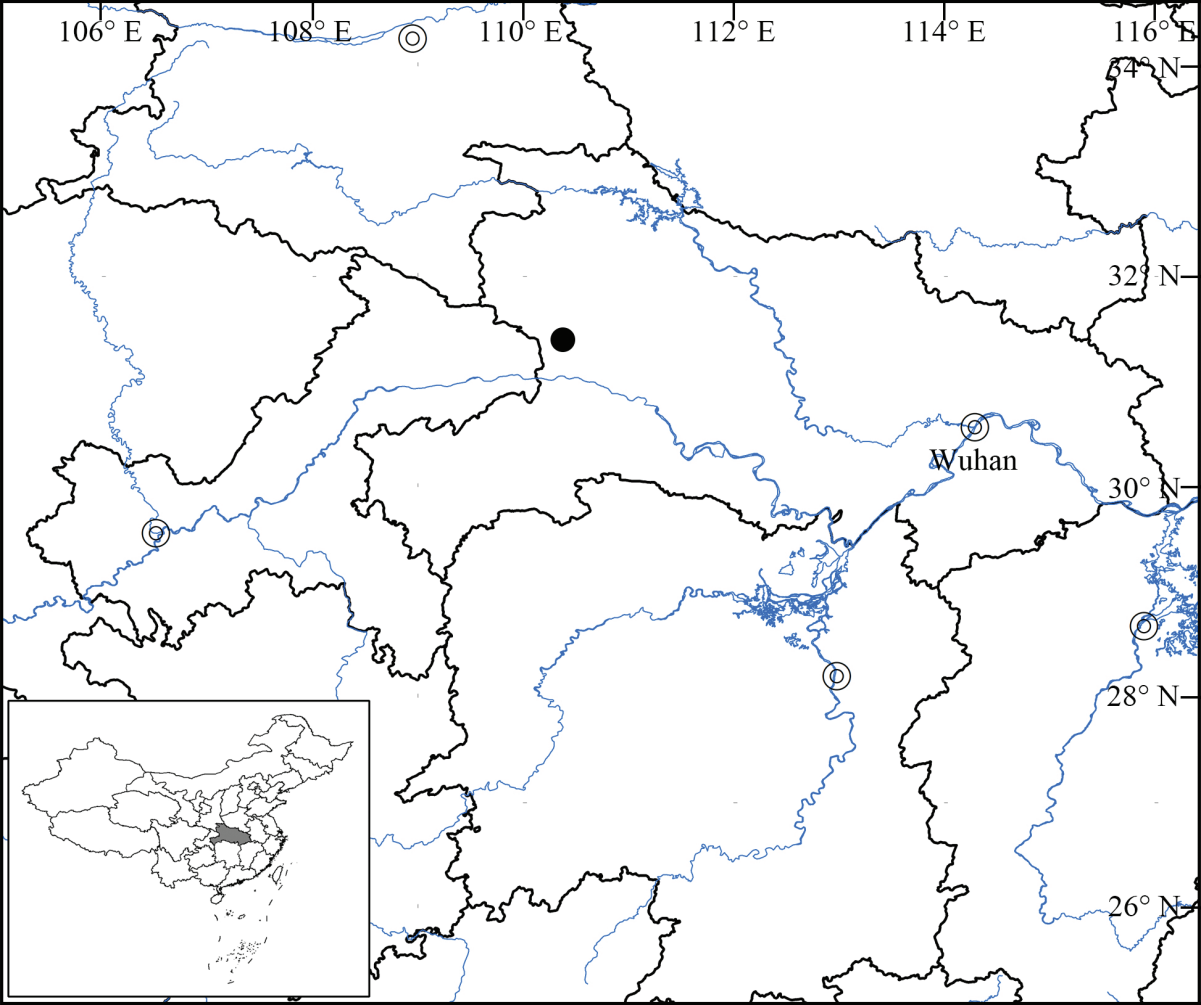
Distribution of *Ligulariamonocephala* sp. nov. (black dot).

This species is currently known only from Shennongjia of Hubei (Fig. 3). It grows in moist forests or on moist cliffs covered by mosses at elevations of between 2681–3026 m above sea level.

#### Additional specimens examined (paratypes).

China. **Hubei**: Shennongjia Forest District, Shennongding Scenic Spot, Badongya, on rocky cliffs, 10 July 1987, *Anonymous 662* (HIB); Shennongjia Forest District, Shennongding Scenic Spot, Shennonggu valley, on moist cliffs covered by mosses, 31°26'42.95"N, 110°15'49.88"E, 3026 m a.s.l., 22 September 2020, *L. Wang*, *X.Q. Guo & Q.E. Yang 4216* (IBSC); ibid., on cliffs, 2852 m, 9 August 2008, *X.L. Yu et al. 080078* (CSFI); Shennongjia Forest District, precise locality unknown, 17 August 2012, *D.G. Zhang 080826018* (JIU); Shennongjia Forest District, precise locality unknown, in rock crevices, 2800 m a.s.l., 2 September 1980, *Z.E. Zhao 1609* (HIB).

## ﻿Discussion

The generic placement of this newly described taxon, which is superficially similar to some species of *Cremanthodium* in the general habit (the gatherings *Anonymous 662*, *D.G. Zhang 080826018*, and *Z.E. Zhao 1609* were, in fact, previously identified on the determination slips as species of *Cremanthodium*), is worthy of detailed remarks. The independent generic status of *Cremanthodium*, the putatively closest ally of *Ligularia*, has been widely accepted in recent checklists or Floras at the national level (e.g. Mathur 1995; Liu 1989; Grierson and Springate 2001; Kress et al. 2003; Liu and Illarionova 2011). Morphologically, the former is generally considered different from the latter by its broadly campanulate or hemispheric (vs. cylindrical or obconic) involucre, although this character is not always applicative in a few species (for example, *C.liangshanicum* L. Wang, C. Ren & Q.E. Yang). Geographically, the former is a high-alpine genus endemic to Sino-Himalayan region with its distribution range locating at 25°–40°N, 75°–104°E (Liu et al. 2002; Wang 2018), while the latter is an Eurasian genus more widely distributed than the former (Liu et al. 1994; Liu et al. 2002). However, the recognition and segregation of *Cremanthodium* has also been repeatedly questioned. It was once considered as an alpine variant (Wulff 1944) or ecotype (Drury 1967) of *Ligularia*. In addition, recent molecular phylogenetic studies focused on the *LCP complex* (*Ligularia*-*Cremanthodium*-*Parasenecio*; Asteraceae, Senecioneae) (Liu et al. 2006; Ren 2012; Ren et al. 2017, 2020) revealed that the two genera are not monophyletic as traditionally defined. They together form three distinct and distantly related clades on chloroplast gene trees, with two clades having the species of the two genera interspersed between each other. The results contradict heavily with Liu’s infrageneric classification system (Liu 1982, 1985) of the two genera established based mainly on morphological characters, but seem to be well correlated with the geographical distributions. Based on the above discussion, the newly reported species, characterizing by having cylindrical involucre, and locating at 31°N, 110°E, is here tentatively placed within *Ligularia* on morphological and geographical grounds.

In the genus *Ligularia*, *L.monocephala* is tentatively assigned to L.sect.Corymbosae (Franch.) Hand.-Mazz. ser.Retusae S.W. Liu due to its palmate-pinnate leaf venation, lanceolate to subulate bract, and cylindrical involucre. Within this series, it resembles *L.phoenicochaeta* (Franch.) Hand.-Mazz. to some extent, but differs mainly by the abaxially purplish red (vs. pale green) leaf blades (Fig. 2C), the erect (vs. cernuous) capitula (Fig. 2E), the cylindrical (vs. hemispheric) involucres (Fig. 2E), and the 5–7 mm (1–2 mm) long pappus (Fig. 2I). *Ligulariamonocephala* is also superficially similar to *L.jamesii* (Hemsl.) Kom. of sect. Ligulariaser.Monocephalae (Nakai) Kitam. and *L.hookeri* (C.B. Clarke) Hand.-Mazz. (those plants with solitary capitula) of sect. Ligulariaser.Ligularia, particularly in the general habit and the solitary capitula. Morphologically, *L.monocephala* differs from *L.jamesii* immediately by the reniform to cordate-reniform (vs. triangular-hastate) leaf blades which are abaxially purplish red (vs. pale green, rarely purplish red) and apically rounded or slightly obtuse (vs. acute or acuminate) (Fig. 2C), the cylindrical (vs. broadly campanulate) involucres (Fig. 2E), the oblong to elliptic (vs. linear-lanceolate) ray laminae which are 6–7 mm (vs. 3–4 mm) wide (Fig. 2H), and the pappus which are as long as or slightly longer than tube of tubular corolla (vs. as long as tubular corolla) (Fig. 2I); and *L.monocephala* differs from *L.hookeri* mainly by the abaxially purplish red (vs. pale green, rarely purplish red) leaf blades (Fig. 2C), the erect (vs. cernuous to horizontal) capitula (Fig. 2E), the oblong to elliptic (vs. linear) ray laminae which are 6–7 mm (vs. 1.5–2 mm) wide (Fig. 2H), and the pappus which are as long as, or slightly longer than, the tube of tubular corolla (vs. as long as tubular corolla) (Fig. 2I). A detailed comparison of the four species is given in Table 1.

**Table 1.** Differences among *Ligulariahookeri*, *L.jamesii*, *L.monocephala* and *L.phoenicochaeta*.

	*L.hookeri*	*L.jamesii*	*L.monocephala*	*L.phoenicochaeta*
Stems	distally white arachnoid and shortly brown pilose	distally white arachnoid-puberulent	densely brownish pilose	distally shortly brown pilose
Basal leaves	leaf blade cordate-sagittate or reniform, abaxially pale green, rarely purplish red; margin triangularly or coarsely dentate, between teeth shortly pilose, apex rounded; palmately veined	leaf blade triangular-hastate, abaxially pale green; margin sharply dentate, apex acute or acuminate; palmately-pinnately veined	leaf blade reniform to cordate-reniform, abaxially purplish red; margin regularly dentate, apex rounded or slightly obtuse; palmately-pinnately veined	leaf blade orbicular-reniform, abaxially pale green; margin regularly triangular-dentate, apex rounded; palmately veined
Capitula	usually solitary, sometimes 2–7(–16) arraged in a raceme; cernuous to horizontal	solitary; erect	solitary; erect	usually solitary, sometimes 2–4 arranged in a lax corymb; cernuous
Involucres	campanulate, 6–8(–10) mm in diam., outside shortly brown pilose or glabrous	broadly campanulate, to 1.5 cm, outside white arachnoid-puberulent	cylindrical, 9–14 mm in diam., outside shortly brownish puberulent	hemispheric, to 24 mm in diam., outside glabrous
Ray florets	lamina linear, 1.5–2 mm wide	lamina linear-lanceolate, 3–4 mm wide	lamina oblong to elliptic, 6–7 mm wide	lamina elliptic or oblong-lanceolate, ca. 2 mm wide
Pappus	brown or pale brown, 6–7 mm long, as long as tubular corolla	pale yellow, 7–8 mm long, as long as tubular corolla	brown in the upper two-thirds, white (distal part) and purplish red (proximal part) in the lower one-third, 5–7 mm long, as long as or slightly longer than tube of tubular corolla	purplish brown, 1–2 mm long, much shorter than tube of tubular corolla
Distribution in China	Shaanxi, Sichuan, Xizang, Yunnan	Jilin, Liaoning, Nei Mongol	Chongqing, Hubei	Yunnan

## Supplementary Material

XML Treatment for Ligularia monocephala
